# Apolipoproteins have a major role in cellular tumor dormancy in triple negative breast cancer: In-silico study

**DOI:** 10.1038/s41598-024-71522-z

**Published:** 2024-10-04

**Authors:** Zaynab El-Gammal, Usama Bakry, Ahmed F. El-Sayed, Toka A. Ahmed, Gehad Atef Oura, Shimaa E. Elshenawy, Nagwa El-Badri, Amin F. Romany, Khaled Amer, Tarek Elnagdy, Osama Mahmoud Azmy, Tarek Taha Ahmed Ali

**Affiliations:** 1 Stem Cells and Regenerative Medicine Branch, Egypt Center for Research and Regenerative Medicine (ECRRM), Cairo, Egypt; 2 Microbial Genetics Department, Biotechnology Research Institute, National Research Centre, Cairo, Egypt; 3 Center of Excellence for Stem Cells and Regenerative Medicine, Zewail City of Science and Technology, Cairo, Egypt; 4 Military Medical Academy, Cairo, Egypt; 5 Reproductive Health Department, Medical Research and Clinical Studies Institute, National Research Centre, Cairo, Egypt

**Keywords:** Breast cancer, Computational biology and bioinformatics

## Abstract

Triple-negative breast cancer (TNBC) lacks estrogen, progesterone, and human epidermal growth factor receptors and has a poor prognosis as it is resistant to chemotherapy. A new treatment option for this type of cancer may be by putting these malignant cells into dormancy. The oocyte’s embryonic milieu presents a unique tumor reversion microenvironment by inducing growth arrest and changing cells’ phenotypes. We conducted an in-silico study to determine the most likely oocyte extract (OE) proteins involved in inducing dormancy using HDock, CluPro, and molecular dynamic (MD) simulation. Results showed low energy scores for complexes between OE proteins and four surface markers: K1C14, CLD3, CLD4, and ITA6. Apolipoprotein A1 (APOA1) and Apolipoprotein C3 (APOC3) showed the highest stability and affinity with these four surface markers: K1C14, CLD3, CLD4, and ITA6. These proteins are involved in key tumor-related pathways such as angiogenesis, proliferation, apoptosis, and migration. This will pave the way for exploring novel therapeutic options to induce dormancy in TNBC cells.

## Introduction

Breast cancer (BC) is the world’s most prevalent cancer, affecting 2.3 million women globally and causing 670,000 deaths in 2022^1^.

Surgery is the gold standard treatment for BC to eradicate the tumor and prevent metastatic spread, followed by adjuvant therapy for decades. However, recently, biomarker and target evaluation have been recognized as neoadjuvant treatments^2^. The treatment plan is greatly dependent on the subtypes of BC. These subtypes are classified based on the presence of three markers: estrogen receptor (ER-positive), progesterone receptor (PR-positive), and human epidermal growth factor receptor 2 (HER2-positive). Triple-negative breast cancer (TNBC) lacks the expression of these three receptors. It accounts for 10–20% of all breast cancer cases and is associated with a high recurrence, distant metastasis, and poor survival outcomes^3^_._ Owing to their biological and clinical heterogeneity, adjuvant therapy for TNBC is of limited effect^4^. Cancer stem cells (CSC) were reported to contribute to poor outcomes and recurrence as they remain dormant and escape the therapeutic effects of chemotherapy and radiation therapy^5^, thus maintaining tumor growth and initiating metastasis_._

New therapeutic approaches are required to specifically target the molecular mechanisms underlying CSC’s oncogenic behavior. A promising approach to avoiding the metastatic potential of these dormant cells is to maintain them in a harmless state, known as the sleeping strategy^6^. This cellular dormancy can be maintained by inhibiting the proliferation pathways, stimulating dormancy-regulating signals, or modulating the cells’ niche. This keeps the cells in a reversible growth arrest and non-proliferative state^6,7^.

However, tumor reversion is hampered by the involvement of different genes and the complexity of the cell reprogramming pathways_._ The embryonic and oocyte environments present unique opportunities for tumor reversion by inducing growth arrest and changing cells’ phenotypes^8^.

The oocyte cytoplasm contains all the necessary elements for successful reprogramming, where oocytes reprogram cancer cells to dormancy in both amphibian and mammalian cells^9–11^. Several proteins present in oocyte extract (OE) may be implicated in the process of dormancy. Among these proteins are human apolipoproteins (APOs), which are classified into 11 subgroups: ApoA, ApoB, ApoC, ApoD, ApoE, ApoF, ApoH, ApoJ, ApoL, ApoM, and ApoO, and most of them are divided into different subtypes (e.g., ApoC is subdivided into ApoC1, ApoC2, ApoC3)^12^. The in-silico study can shed light on the complex interactions between the proteins and their respective receptors. Molecular dynamics (MD) simulation can anticipate the protein complex’s structural changes, stability, and flexibility^13^. This study aims to identify the OE proteins that interact with MDA-MB-231 TNBC cell surface markers to exert the reprogramming effect. This data will be relevant for developing dormancy-promoting drugs using purified OE-enriched factors to limit the malignant behavior of TNBC.

## Material and methods

**Fig. 1:**
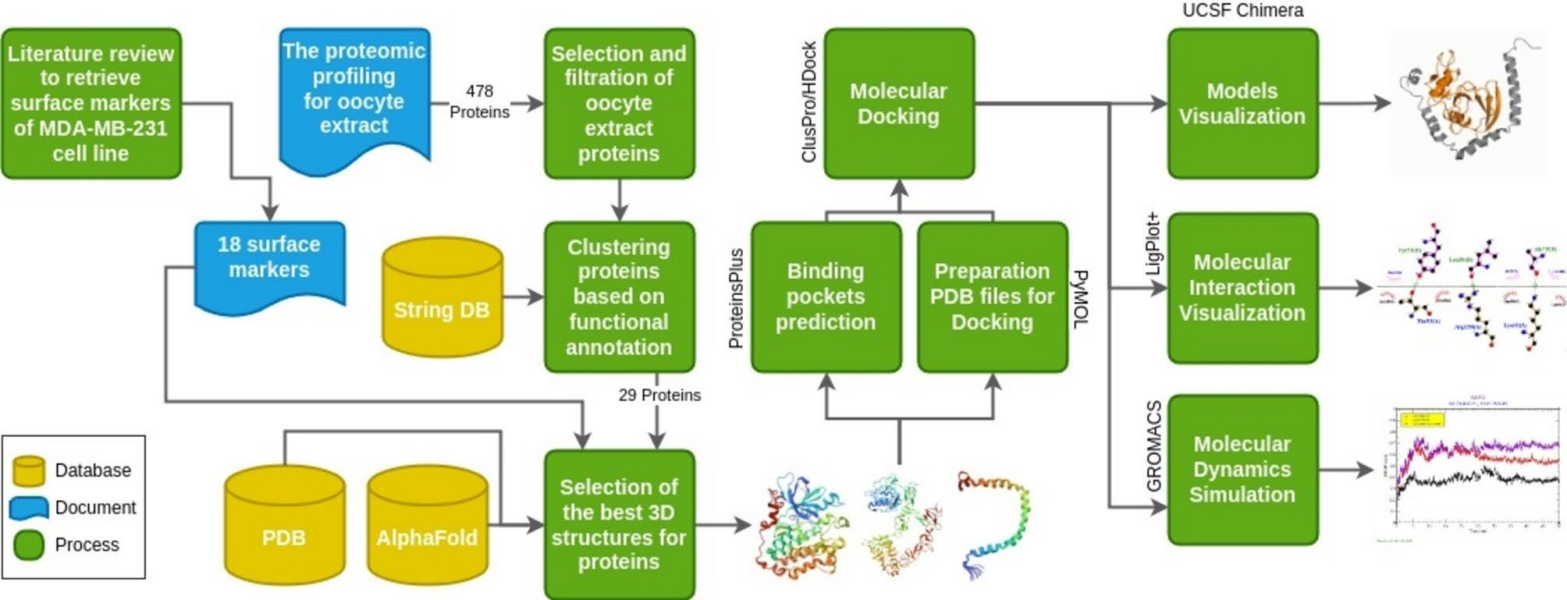
Diagrammatic representation of data retrieval, selection, and filtration, and clustering of the best 3D structure for oocyte extract proteins and MDA-MB-231 surface markers (String DB: String database; PDB: Protein Data Bank).

We used an in-silico approach to predict and confirm the protein design through molecular docking and MD simulations. This study was approved by the Institutional Review Board of the Egypt Center for Research and Regenerative Medicine (approval #3/06-2022). Figure 1 represents a diagrammatic workflow of data retrieval, and the selection, filtration process, and clustering of the best 3D structure proteins and surface markers present in the OE and MDA-MB-231 cell lines.

### Retrieval of surface markers of the MDA-MB-231 cell line

All reported surface markers on TNBC cell lines (MDA-MB-231) were retrieved from the available English literature until March 2024. Table 1 lists all the surface markers with their gene names, UniProt, and 3D structure accessions.

**Table 1:** List of MDA-MB-231 surface markers with their gene names, UniProt accession, and 3D structure accession.

Protein	Gene name	UniProt accession	3D Structure accession
Claudin-3	CLD3	O15551	AF-O15551-F1
Claudin-4	CLD4	O14493	AF-O14493-F1
CD44 antigen	CD44	P16070	4PZ3
Proliferation marker protein Ki-67	KI67	P46013	5J28
Epithelial cell adhesion molecule	EPCAM	P16422	AF-P16422-F1
Epidermal growth factor receptor	EGFR	P00533	3QWQ – 3W32
E-Cadherin	CADH1	P12830	4ZT1
N-Cadherin	CADH2	P19022	AF-P19022-F1
Integrin alpha-V/beta-3	αVβ3	P05106 , P06756	3IJE
Intercellular adhesion molecule 1	ICAM1	P05362	1IAM
Cell surface glycoprotein MUC18	MUC18	P43121	AF-P43121-F1
Cell surface glycoprotein MUC18		P43121	6LYN
Keratin, type I cytoskeletal 14	K1C14	P02533	AF-P02533-F1
Integrin alpha-6	ITA6	P23229	AF-P23229-F1
Urokinase plasminogen activator surface receptor	UPAR	Q03405	AF-Q03405-F1
Vimentin	VIME	P08670	AF-P08670-F1
Programmed cell death 1 ligand 1	PD1L1	Q9NZQ7	3BIS
Leukocyte surface antigen CD47	CD47	Q08722	7WN8
Signal-induced proliferation-associated protein 1	SIPA1	Q96FS4	AF-Q96FS4-F1

### Selection and filtration of OE proteins

Following the proteomic profiling of OE reported in our earlier study^14^, we eliminated reversed proteins, isoforms, duplicates, uncharacterized proteins, and mutant proteins during the first phase of the protein filtration process. We then filtered the chosen proteins by protein function and sequence similarity by performing multiple sequence alignments using Multalin^15^. We clustered proteins based on their functions using the String database^16^. Table 2 displays the selected proteins with their gene names, UniProt, and 3D structure accessions.

**Table 2:** List of oocyte extract proteins with their gene names, UniProt accession, and 3D structure accession.

Protein	Gene name	UniProt accession	3D Structure accession
Serotransferrin	TRFE	P02787	1A8E
NAD(P)H dehydrogenase [quinone] 1	NQO1	P15559	1D4A
Vitamin D-binding protein	VTDB	P02774	1KW2
Zinc-alpha-2-glycoprotein	ZA2G	P25311	1T80
Apolipoprotein C-III	APOC3	P02656	2JQ3
Thyroxine-binding globulin	THBG	P05543	2XN6
Alpha-1-acid glycoprotein 2	A1AG2	P19652	3APU
Alpha-1-acid glycoprotein 1	A1AG1	P02763	3KQ0
Apolipoprotein A-I	APOA1	P02647	3R2P
Corticosteroid-binding globulin	CBG	P08185	4C41
Ubiquitin carboxyl-terminal hydrolase isozyme L1	UCHL1	P09936	4DM9
Ceruloplasmin	CERU	P00450	4ENZ
Plasma protease C1 inhibitor	IC1	P05155	5DU3
Myosin-14	MYH14	Q7Z406	5I4E
Angiotensinogen	ANGT	P01019	5M3Y
Retinol-binding protein 4	RET4	P02753	5NU7
NAD-dependent protein deacetylase sirtuin-6	SIR6	Q8N6T7	5Y2F
ATP-binding cassette sub-family F member 1	ABCF1	Q8NE71	5ZXD
Beta-2-glycoprotein 1	APOH	P02749	6V06
Transthyretin	TTHY	P02766	7EJQ
Msx2-interacting protein	MINT	Q96T58	7Z1K
Apolipoprotein A-II	APOA2	P02652	AF-P02652-F1
Leucine-rich alpha-2-glycoprotein	A2GL	P02750	AF-P02750-F1
Alpha-2-HS-glycoprotein	FETUA	P02765	AF-P02765-F1
Alpha-1B-glycoprotein	A1BG	P04217	AF-P04217-F1
Clusterin	CLUS	P10909	AF-P10909-F1
Lumican	LUM	P51884	AF-P51884-F1
Transmembrane protein 198	TM198	Q66K66	AF-Q66K66-F1
Elongin-A2	ELOA2	Q8IYF1	AF-Q8IYF1-F1

### Preparation of selected proteins

Domain coverage, lack of mutations and gaps, resolution less than 3 Å, and the experimental method of x-ray diffraction determine the best selection of the 3D structure for MDA-MB-231 surface markers and the chosen proteins of the OE. We applied the Protein Data Bank (PDB) for all proteins with a 3D structure and utilized the AlphaFold Protein Structure Database for proteins with 3D structures that did not meet the selection criteria. PyMOL removed identical chains, water particles, and small and co-crystalized molecules to prepare all protein and surface marker structures^17^.

### Molecular docking and interaction

DoGsite maps the possible binding pockets based on descriptor calculations such as depth, surface, and volume. The DoGSite Scorer web server, a strong tool for investigating potential binding pockets, identified the possible binding pockets of receptors and proteins. Furthermore, the support vector machine (SVM) method estimated the druggability score. This score is graded from 0 to 1, where higher values are the potential pockets for the main binding sites^18,19^. The ClusPro server (https://cluspro.org), the most common tool used for protein–protein docking, performed the MD and the Fast Fourier Transform (FFT) correlation^20^. For template-based modeling and ab initio-free docking, we applied the HDock server as a confirmatory docking tool based on a hybrid algorithm^21^. LigPlot + and UCSF Chimera generated the protein–protein interaction diagrams and the complex visualizations, respectively^22,23^.

### Molecular dynamic simulation

We applied the GROMACS 5.1.2 software for MD simulations with the Gromos96 53a7 force field to investigate the stability and flexibility of the proteins. To set up the simulation systems, all proteins were placed in a cubic water box measuring 90.0 × 90.0 × 90.0 nm. The simple point-charge water model represented the water molecules. Sodium ions were added to neutralize the entire protein system. To ensure a well-behaved system and resolve any steric clashes or geometric irregularities in the solvated protein system, we conducted an energy minimization step. Subsequently, the minimized system underwent equilibration in two phases: a constant number of particles, volume, and temperature (NVT) and a constant number of particles, pressure, and temperature (NPT). The equilibration process lasted for 1000 picoseconds (ps). Following the equilibration phase, the protein systems, that are now well-equilibrated, entered the production run. This run lasted for 50 ns (ns) under a temperature of 300 K (Kelvin) and a pressure of one atmosphere. To provide valuable insights into the dynamic behavior, stability, and intermolecular interactions of the proteins during the production run, we analyzed the dynamic behavior and stability of each protein using the root mean square deviation (RMSD), the radius of gyration (Rg), the root mean square fluctuation (RMSF), and the solvent accessible surface area (SASA). RMSD ascertains the stability of the protein structures. The minimum value of the RMSD (~ 0.2–0.5 ~ 0.5 nm) means that the protein complex is in a good stability state^24^. The Rg outlines the overall shape of the protein complexes, and RMSF assures the flexibility of amino acid residues during the simulation^25^. Next, the SASA analysis was conducted to understand the protein folding dynamics. We then assessed the stability of the complexes by analyzing the intra- and inter-molecular hydrogen bonds.

## Results

### Selection and filtration of oocyte extract proteins

The proteomic profiling of OE revealed the expression of 478 proteins. Based on the selection and filtration process of these proteins, 29 proteins were shortlisted for downstream molecular docking experiments (Fig. 1).

### Molecular docking for MDA-MB-231 surface markers and OE proteins

**Fig. 2:**
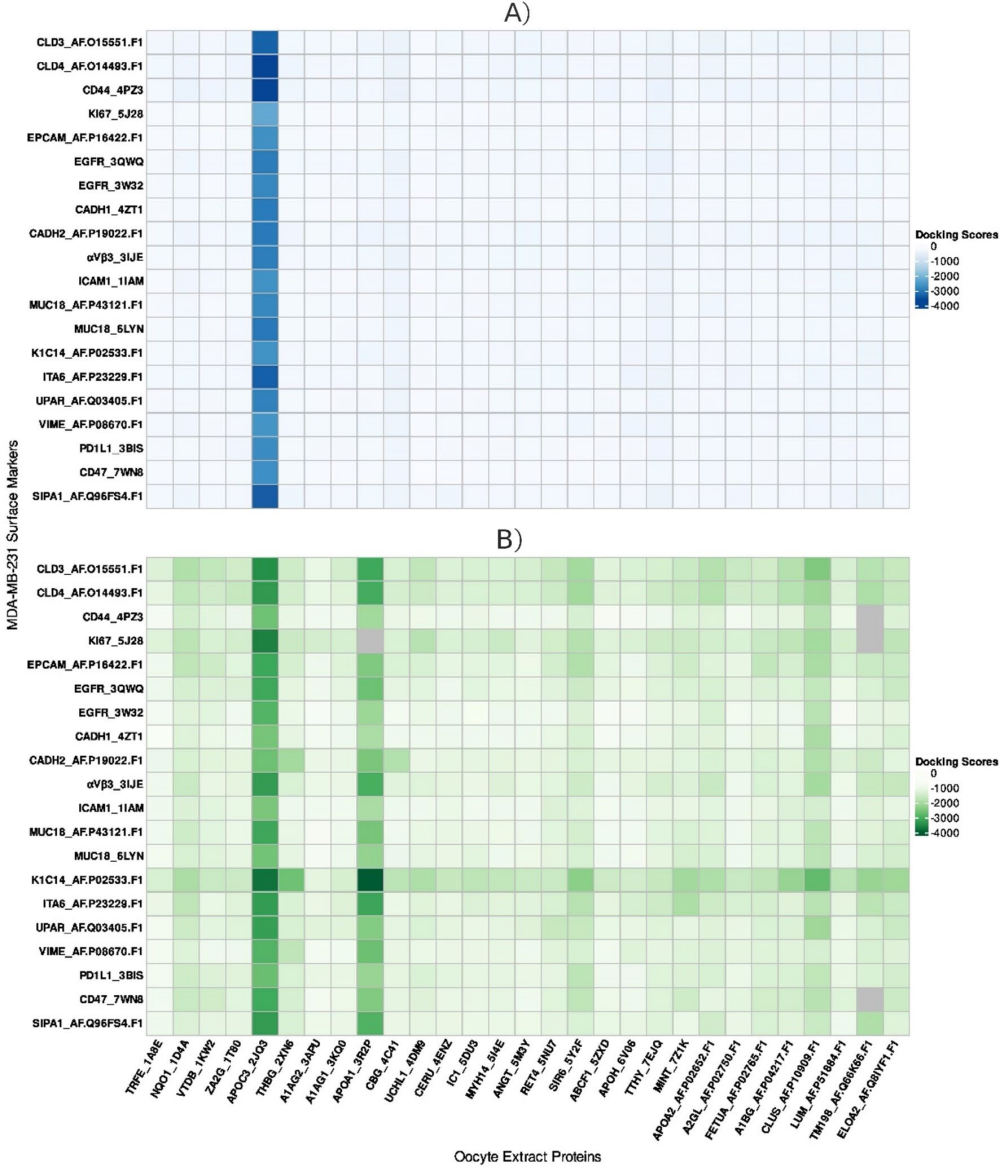
The heat map of energy scores for all 29 OE proteins and surface markers complexes by both HDock (**A**) and ClusPro (**B**) servers.

**Fig. 3:**
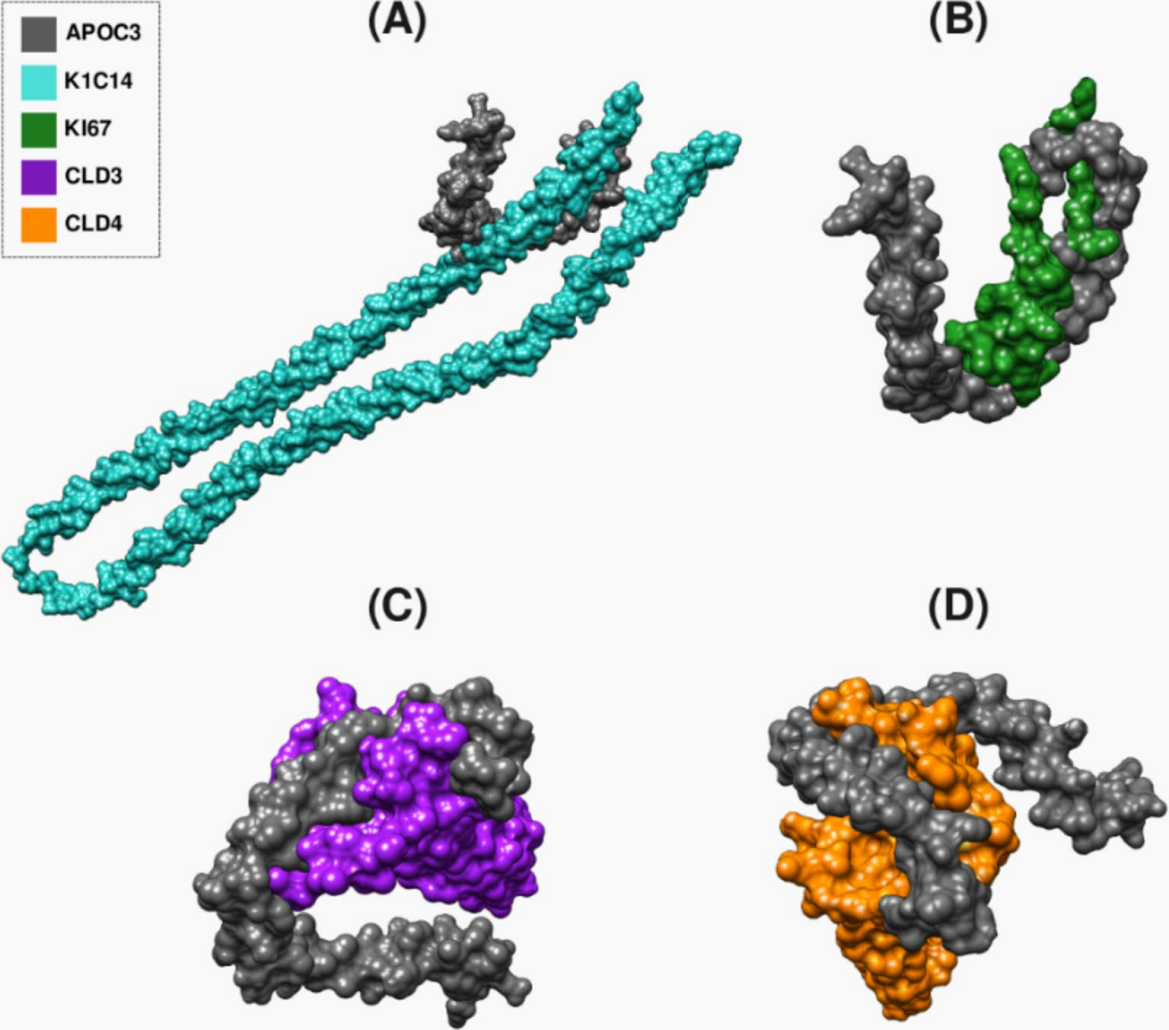
The complex interaction between APOC3 and (**A**) K1C14, (**B**) KI67, (**C**) CLD3, and (**D**) CLD4 surface markers visualized by PyMol.

**Fig. 4:**
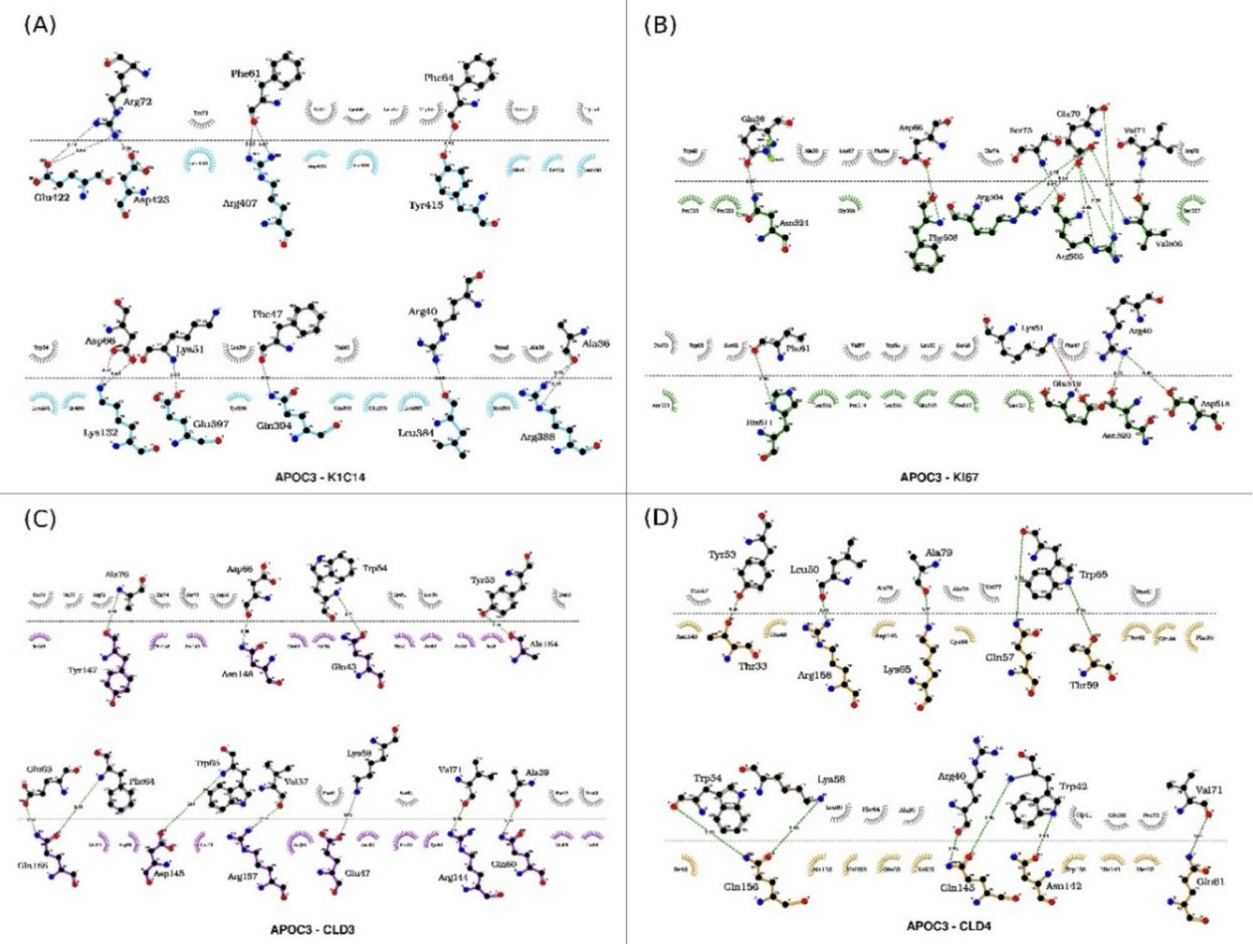
The 2D interactions between APOC3 and (**A**) K1C14, (**B**) KI67, (**C**) CLD3, and (**D**) CLD4 surface markers using LigPlot( +).

**Fig. 5:**
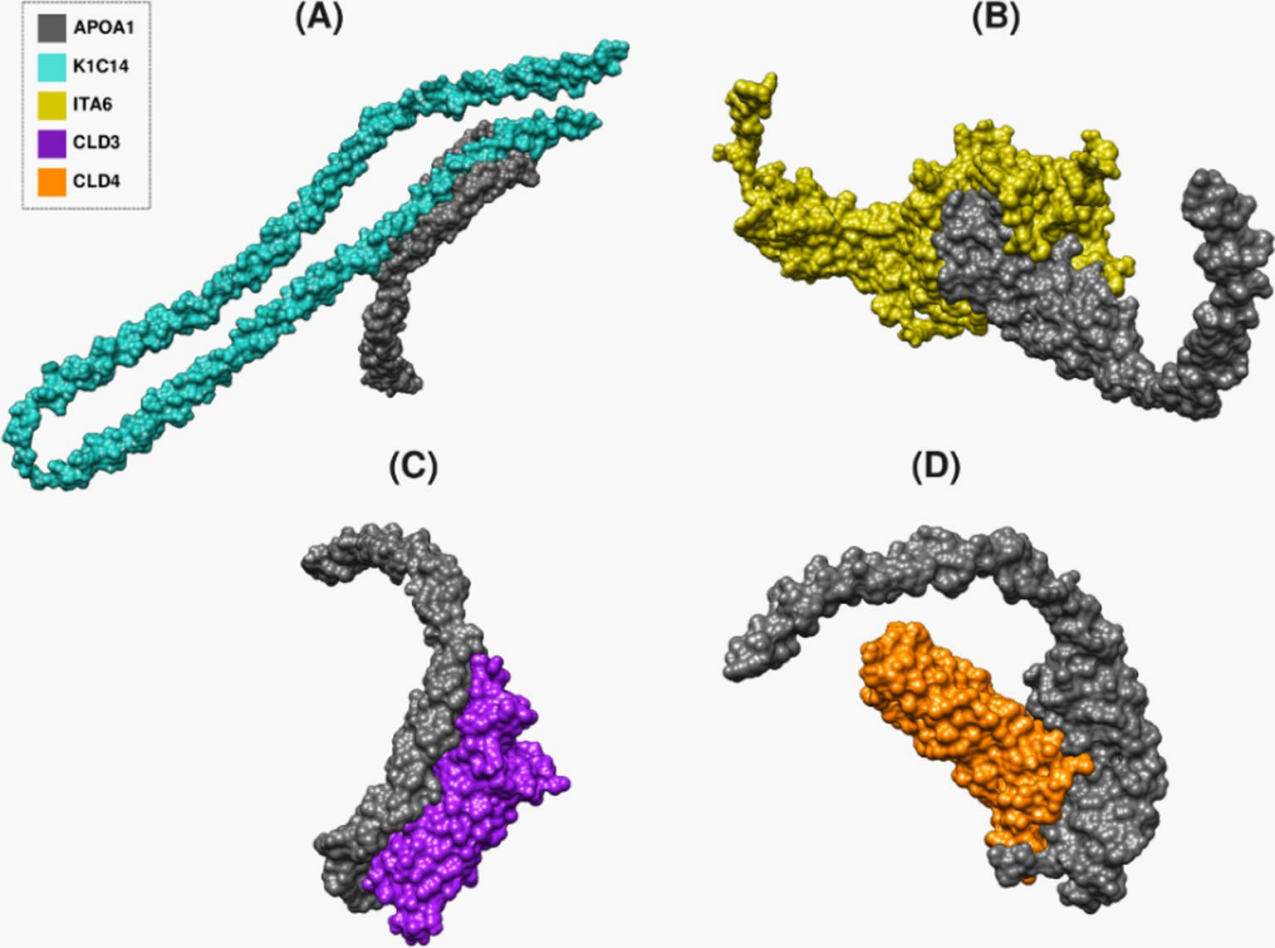
The complex interaction between APOA1 (**A**) K1C14, (**B**) ITA6, (**C**) CLD3, and (**D**) CLD4 surface markers visualized by PyMol.

**Fig. 6:**
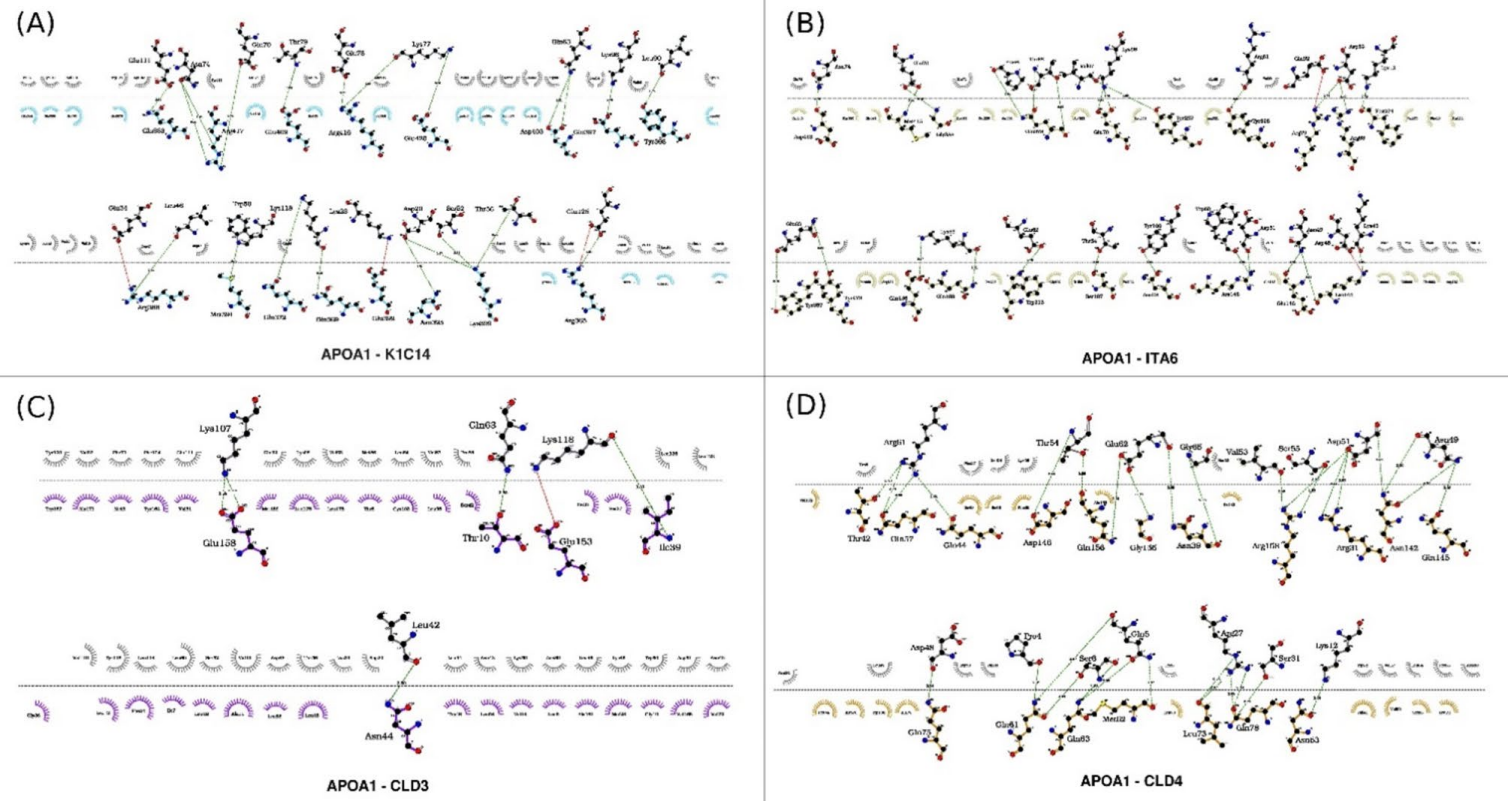
The 2D interactions between APOA1 (**A**) K1C14, (**B**) ITA6, (**C**) CLD3, and (**D**) CLD4 surface markers using LigPlot( +).

Using the ClusPro web server, the heatmap results showed low energy scores for all complexes between the 29 OE proteins and MDA-MB-231 surface markers identified by the docking experiments, except 4 complexes (APOA1-KI67, TM198-CD47, TM198-KI67, and TM198-CD44) (Fig. 2). In addition, the docking results displayed that APOC3 and APOA1 had the lowest energy scores (Apendix1) with the following surface markers: K1C14 (− 3751.6, − 4011.6, respectively) (Figs. 3A, 4A, 5A, 6A, respectively), CLD3 (− 3461.6, − 3087.7, respectively) (Figs. 5C, 6C), CLD4 (− 3301.1, − 3031.7, respectively) (Figs. 5D, 6D), and ITA6 (− 3138.8, − 3265.4, respectively). Also, APOC3 showed low energy scores with KI67 (− 3628.8) (Figs. 2B, 3B, and 4B), while APOA1 exhibited low energy scores with ITA6 (− 3138.8) (Figs. 2B, 5B, and 6B). Hdock’s analysis confirmed that APOC3 demonstrated the lowest energy scores with CLD3 (Figs. 2A, 3C, and 4C) (− 3301.2) and CLD4 (− 3625.6) (Figs. 2A, 3D, and 4D).

### Molecular dynamics simulation

**Fig. 7:**
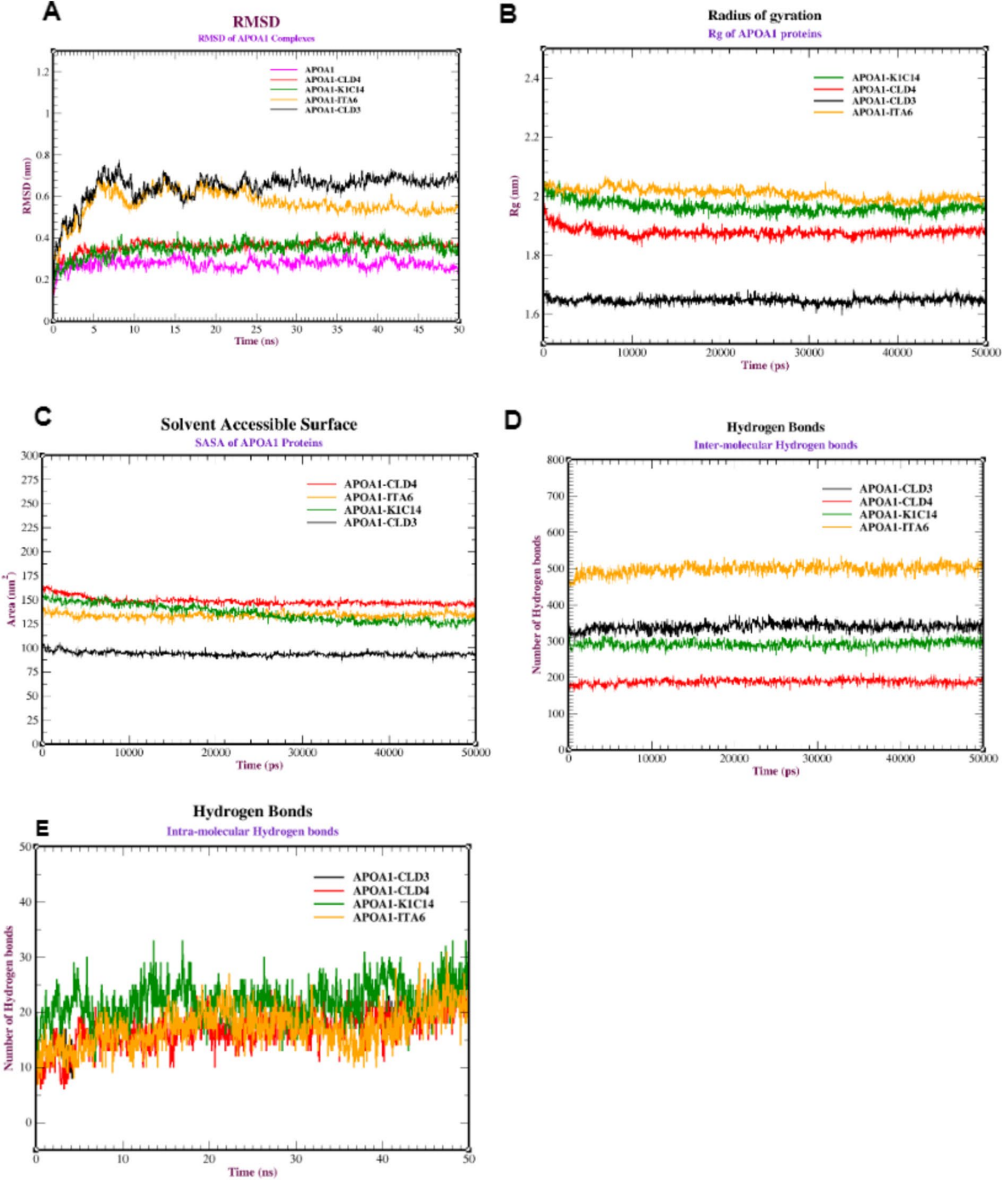
MD simulation of APOA1 protein against the receptors (CLD3, CLD4, K1C14, and ITA6): (**A**) RMSD of the backbone conformation, (**B**) Radius of gyration, (**C**) Solvent-accessible surface area (SASA) analysis, (**D**) Intermolecular hydrogen bonds, and (**E**) Intramolecular hydrogen bonds in APOA1 protein against the various receptors (CLD3, CLD4, K1C14 and ITA6).

Based on the docking of the 29 OE proteins with the MDA-MB-231 surface markers, dynamics simulations were performed to investigate the behavior and stability of protein complexes at the molecular level. The several analyses performed to assess the stability and dynamics of the APOA1 complexes with the following receptors (K1C14, CLD4, ITA6, and CLD3) revealed the following:

The RMSD for the APOA1-K1C14 complex is the highest stable complex between 0.20 and 0.3 nm), followed by the APOA1-CLD4 complex between 0.30 and 0.35 nm, the APOA1-ITA6 complex between 0.4 and 0.60 nm, and the APOA1-CLD3 complex between 0.4 and 0.70 nm. The RMSD of the APOA1-K1C14 complexes achieved stability after 10 ns with RMSD of 0.30 nm. Similarly, APOA1-CLD4 complexes had an average RMSD of 0.35 nm after 15 ns. From 0 to 50 ns, excessive variation in the RMSD of the APOA1-ITA6 and APOA1-CLD3 complexes was observed due to the extreme deviation of the proteins from their initial positions. The APOA1 protein exhibited a similar pattern of stability and conformation change with the same four surface markers during the simulation. After 10 ns, the complex of APOA1 with the surface markers achieved a stable conformation with an average RMSD of (0.30, 0.35, 0.60, and 0.66 nm) for K1C14, CLD4, ITA6, and CLD3) respectively (Fig. 7A).

Rg ranges between 1.66 and 1.70 nm for the APOA1-CLD3 complex. While Rg starts at 2.00 nm and decreases to 1.90 nm in the final 50 ns of the simulation for the APOA1-CLD4 complex. This decrease in Rg indicates a more compact and potentially more stable structure. In contrast, APOA1-K1C14 and APOA1-ITA6 complexes show stabilization with relatively constant average Rg values of 2.00 nm, and 2.05 nm, respectively (Fig. 7B).

SASA values range from 90 to 110 nm^2^ for the APOA1-CLD3 complex, and 135 to 165 nm^2^ for the APOA1-CLD4, APOA1-K1C14, and APOA1-ITA6 complexes. While the APOA1-CLD3 complex exhibited a relatively consistent total area ranging from about 90 to 110 nm^2^. On the other hand, APOA1-CLD4, APOA1-K1C14, and APOA1-ITA6 complexes had larger surface areas, ranging from about 135 to 165 nm^2^. Typically, an increased value of protein SASA during the simulation indicates structural relaxation and, consequently, reduced protein stability (Fig. 7C).

There were varying degrees of hydrogen bond interactions between the APOA1 core protein and the different receptor complexes. The APOA1 complexes with (ITA6, CLD3, K1C14, and CLD4) formed a range of (450–500), (300–330), (280–300) and (190–225) inter-molecular hydrogen bonds, respectively, which fluctuated during the simulation (Fig. 7D). While the APOA1-K1C14 complex formed the highest number of intra-molecular hydrogen bonds (15–35 bonds), followed by ITA6 (10–25 bonds) and (5–20 bonds) for both CLD3 and CLD4 (Fig. 7E).

**Fig. 8:**
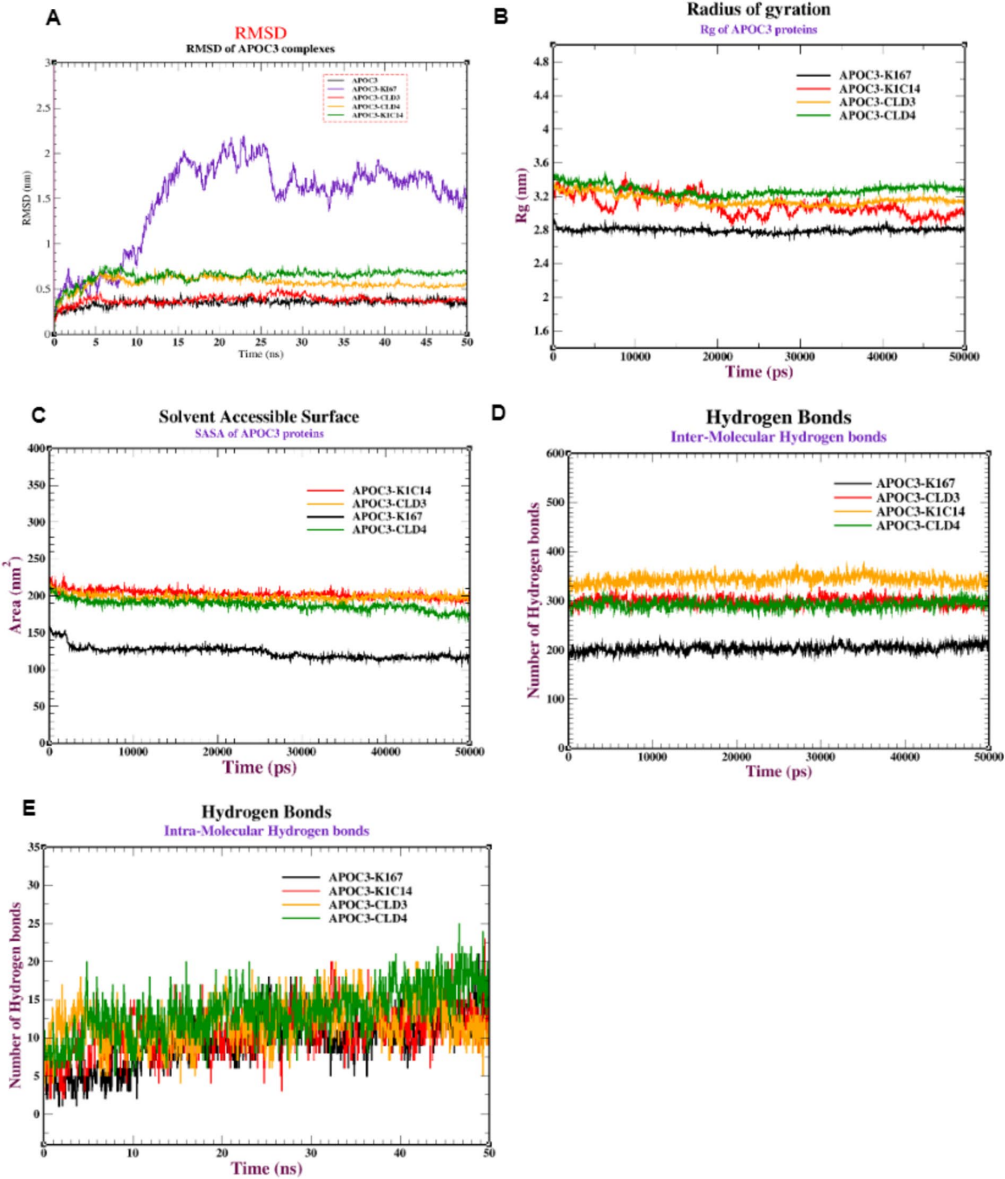
MD simulation of APOC3 protein against the receptors (CLD3, CLD4, K1C14 and KI67): (**A**) RMSD of the backbone conformation, (**B**) Radius of gyration, (**C**) Solvent-accessible surface area (SASA) analysis, (**D**) Intermolecular hydrogen bonds, and (**E**) Intramolecular hydrogen bonds in APOC3 protein against the various receptors (CLD3, CLD4, K1C14 and ITA6).

In addition, MD revealed that the stability and dynamics of the APOC3 protein are variable with the following receptors (K1C14, CLD4, KI67, and CLD3). The RMSD for the APOC3-CLD3 complex is the highest stable complex between 0.22 and 0.35 nm, followed by the APOC3-CLD4 complex between 0.50 and 0.60 nm, then the APOC3-K1C14 complex (between 0.4 and 0.65 nm) and the APOC3-KI67 complex (between 0.5 and 2.0 nm). The RMSD of the APOC3-CLD3 complexes achieved stability after 10 ns, with an RMSD of 0.35 nm. Similarly, APOC3-CLD4 and APOC3-K1C14 complexes had an average RMSD of 0.50 nm after 15 ns. The APOC3-KI67 complex exhibited a higher pattern of RMSD changes during the simulation. After 10 ns, the RMSD values of APOC3-KI67 increased gradually to 2.0 nm. Therefore, the surface markers achieved a stable conformation with an average RMSD of 0.30, 0.55, and 0.60 nm for CLD3, CLD4, and K1C14 respectively (Fig. 8A). Also, the Rg values of the APOC3 complexes range between 1.75 and 3.50 nm.

The Rg of the APOC3-KI67 complex starts at 2.80 nm and is still stable until the end of the simulation. Similarly, complexes APOC3-CLD4 and APOC3-CLD3 show stabilization with relatively constant average Rg values of 3.30 nm and 3.40 nm, respectively (Fig. 8B). The SASA values range from 130 to 145 nm^2^ for the APOC3- KI67 complex and 180 to 230 nm^2^ for the APOC3-CLD4, APOC3-K1C14, and APOC3-CLD4 complexes (Fig. 8C).

There were varying degrees of hydrogen bond interactions between the APOC3 and APOA1 core proteins and the different receptor complexes. The inter-molecular hydrogen bonds of APOC3 complexes with (KI67, CLD3, K1C14, and CLD4) formed a range of (180–220), (280–310), (320–350), and (290–310) respectively, which fluctuated during the simulation (Fig. 8D). In terms of the intra-molecular hydrogen bonds, the APOA1-CLD4 complex formed the highest number of hydrogen bonds (8–20 bonds), followed by CLD3 (6–18 bonds) and (3–15 bonds) for both KI67 and K1C14 (Fig. 8E).

## Discussion

Despite the advances in health care and biotechnology, TNBC remains a challenging dilemma in cancer treatment. OE may open a new hope for women with this aggressive disease and pave the way to a new and innovative approach to the management of cancer.

The interaction of OE proteins and cell receptors that could lead to cell dormancy and oncogenic reversion is complex and perplexing, as we identified 478 proteins through proteomic profiling of OE. Nevertheless, after conducting functional and enrichment pathway analyses, the most active players out of these proteins were probably only twenty-nine. The other proteins were reversed proteins, isoforms, duplicates, uncharacterized proteins, or mutant proteins.

The 29 selected proteins are involved in angiogenesis, apoptosis, proliferation, reactive oxygen species scavenging, tissue remodeling, migration, cancer growth, and chemotherapeutic resistance. However, two proteins showed the highest stability, affinity, and strong interaction with three surface markers on the MDA-MB-231 cell line. These proteins were APOC3 and APOA1, and the three surface markers were K1C14, CLD3, and CLD4. Also, HDock analysis confirmed that these complexes had a relatively compact and stable structure throughout the simulation and advocated that they undergo a conformational change or structural rearrangement during the simulation. These two proteins belong to the APO family. APOs are a group of specialized proteins that function as lipid carriers and cofactors for enzymes and ligands of the cell membrane receptor. He Y and colleagues reviewed the different tumorigenic pathways of APO for different types of cancers^12^.

In our study, APOA1 and APOC3 in the human OE showed the highest affinity and stability with MDA-MB-231 cell line receptors. These proteins showed high affinity and stability for CLD3, CLD4, CK14, KI-67, and ITA6 receptors in MDA-MB-231. CLDN3 is involved in the cell–cell interaction, acting as an adhesion protein^26^. CLDN4 reduces the EMT and hence the cells’ migration potential^27^. CK14 maintains the myoepithelial phenotype and resists environmental mechanical stress^28^. KI-67 is involved in cellular proliferation and adaptation to the environment^29^. Whereas ITA6 maintains the stemness of breast cancer cells, thereby stimulating tumor initiation and metastasis^30^. Also, APOA1 has been reported to inhibit the migration and proliferation of MDA-MB-231 cells by controlling the epithelial-to-mesenchymal transition (EMT) process and orchestrating cholesterol metabolism^31^. Despite APOC3’s high affinity and stability, it did not show direct activity with the MDA-MB-231 cell line. However, APOC3 is involved in the cholesterol metabolism pathway, which is highly related to the tumorigenicity of the MDA-MB-231 cell line.

The affinity of proteins for surface markers may not necessarily relate to the activity of these proteins. We assembled all reported pathways of the different APOs with MDA-MB-231 concerning tumorigenicity (Appendix 2). Although APOJ in the MDA-MB-231 cell line increases cell proliferation, invasion, and migration and decreases cell apoptosis, it shows low affinity for MDA-MB-231 surface markers^32^. Also, two other APOs (APOA2 and APOH) in the OE contributed to the modulation of tumorigenicity as cholesterol metabolism, angiogenesis, and apoptosis, respectively^33^, had no reported crosstalk with the MDA-MB-231 cell line.

Although only two proteins showed high affinity and stability with the aforementioned cell receptors, the interaction of other proteins in the OE and surface receptors on the MDA-MB-231 cell line shows variable affinity and stability and therefore cannot be dismissed, as shown in Appendix I.

Some of these proteins are pertinent to the TNBC angiogenesis process, such as leucine-rich alpha-2-glycoprotein (A2GL) and angiotensinogen (ANGT), by different mechanisms. A2GL disrupts the homeostatic TGF-b pathway and destabilizes the interaction between pericytes and endothelial cells^34^, and ANGT is a precursor of angiotensin II (Ang II) that stimulates the expression of vascular endothelial growth factor (VEGF)^12^.

Also, some OE proteins are involved in TNBC cancer proliferation, such as ZA2G, CBG, SIR6, and NQO1, through different mechanisms. CBG induces the expression of Receptors for Activated C Kinase 1 (RACK1(^35^, CLU stimulates HIF‐1α^36^, SIR6 increases OXPHOS and intracellular calcium concentration^37^, NQO1 acts as a ROS scavenger and modulates glycolytic reprogramming^38^, and ZA2G stimulates the proliferation of the MDA-MB-231 cell line without affecting the expression of apoptosis or differentiation genes^39^.

On one hand, apoptosis is induced by UCHL1, VTDB, TRFE, and MINT in the OE. UCHL1 induces G0/G1 cell cycle arrest^40^, VTDB upregulates the pro-apoptotic genes and downregulates the anti-apoptotic genes^41^, and TRFE imports iron into TNBC cells, whose accumulation promotes cell death^42^. MINT increases phosphorylated extracellular signal-regulated kinase^43^. On the other hand, apoptosis is reduced by A2GL and NQO1. CLU plays a chaperone-like role^44^, A2GL binds to cytochrome c^41^, and NQO1 protects the p53 tumor suppressor protein and detoxifies the quinone metabolites^42^.

TNBC migration is induced by FETUA, A1BG, ANGT, UCHL1, A2GL, and SIR6 by different mechanisms. FETUA acts as a chemo-attractant inducing the adhesion of TNBC to the endothelial micro-vessels of TNBC cells^45^, A1BG stimulates the immune evasion through platelet activation, aggregation, and degranulation^46^, ANGT is the precursor of AngII that induces the adhesion to endothelial cells and upregulates the matrix metalloproteinases^47^, UCHL1 stabilizes TGFβ receptor I (TGFβR1) inducing TGF-β mediated epithelial-to-mesenchymal transition^48^, A2GL induces TGF-β/Smads signaling pathway^49^, SIR6 induces MMP-9 leading to the degradation of the ECM^50^, IC1 inhibits complement C1s and C1r, kallikrein, and coagulation factor XIIa^50^, NQO1 activates AMPK and AKT/mTOR signaling pathways leading to the glycolytic reprogramming (34), and CERU increases glycolysis and decreases the tumor immune cell infiltration^51,52^. However, migration is inhibited by A1AG1 and LUM in the oocyte extract. A1AG1 decreases the micro-vessels’ solute permeability^53^, and LUM downregulates hyaluronan synthase expression that regulates the EMT process^54^.

The disadvantage of this approach is that it does not kill dormant cancer cells, and the clinical outcome would be a minimal residual disease requiring life-long therapy, which is associated with multiple challenges such as patient compliance, cost, and toxicity (Fig. 3A). The effectiveness of dormancy maintenance is also a matter of concern, as not all cells may respond. Some dormant cells are slow-cycling; thus, the tumor mass will eventually grow.

## Conclusion

The identification of the interaction between MDA-MB-231 cell line receptors and human OE proteins gives insight into their role in the reprogramming of MDA-MB-231. APOs are most likely the proteins involved in tumor dormancy induction. These proteins are involved in key tumor-related pathways such as angiogenesis, proliferation, apoptosis, and migration. This paves the way for the exploration of novel therapeutic strategies aimed at inducing dormancy in TNBC.

## Supplementary Information

Supplementary Information.

## Data Availability

Data is provided within the manuscript and supplementary information files.
