# Supplementary material for: Apolipoproteins have a major role in cellular tumor dormancy in triple negative breast cancer: In-silico study
Source: Sci Rep. 2024 Oct 4;14:23146. doi: 10.1038/s41598-024-71522-z (PMC11452491; doi:10.1038/s41598-024-71522-z)

**Supplementary data**

**Appendix 1:** Docking Scores using ClusPro

|  |  | **CLD3** | **CLD4** | **CD44** | **KI67** | **EPCAM** | **EGFR** | | **CADH1** | **CADH2** | **αVβ3** | **ICAM1** | **MUC18** | | **K1C14** | **ITA6** | **UPAR** | **VIME** | **PD1L1** | **CD47** | **SIPA1** |
| --- | --- | --- | --- | --- | --- | --- | --- | --- | --- | --- | --- | --- | --- | --- | --- | --- | --- | --- | --- | --- | --- |
|  |  | **AF-O15551-F1** | **AF-O14493-F1** | **4PZ3** | **5J28** | **AF-P16422-F1** | **3QWQ** | **3W32** | **4ZT1** | **AF-P19022-F1** | **3IJE** | **1IAM** | **AF-**  **P43121-F1** | **6LYN** | **AF-P02533-F1** | **AF-P23229-F1** | **AF-Q03405-F1** | **AF-P08670-F1** | **3BIS** | **7WN8** | **AF-Q96FS4-F1** |
| **APOC3** | **2JQ3** | -3461.6 | -3301.1 | -2623.4 | -3628.8 | -3101.3 | -3085.1 | -2893.3 | -2545 | -2638.2 | -3274.4 | -2525.4 | -3110.6 | -2602 | -3751.6 | -3265.4 | -3213.1 | -2902.1 | -2687.3 | -3066.4 | -3274 |
| **APOA1** | **3R2P** | -3087.7 | -3031.7 | -2085.5 | NaN | -2448.2 | -2639.4 | -2148.9 | -1969.5 | -2526.3 | -2987.1 | -1998 | -2553.9 | -2248.5 | -4011.6 | -3138.8 | -2409.9 | -2642 | -2191.6 | -2429.2 | -2912.2 |
| **CLUS** | **AF-P10909-F1** | -2456.7 | -2132 | -1814.5 | -2077.8 | -1989.8 | -1666.5 | -1794.7 | -1991.4 | -1900.1 | -2041.2 | -1407.4 | -1754.2 | -1762 | -2742.7 | -1802.6 | -2118.2 | -1380 | -1706.7 | -1810.4 | -1571.5 |
| **SIR6** | **5Y2F** | -2045.5 | -2088.5 | -1376.3 | -1862.7 | -1909.3 | -1557.1 | -1282.8 | -1345.4 | -1357 | -1510.9 | -1250.6 | -1424.2 | -1357 | -2337.3 | -1819.5 | -1630 | -1369.4 | -1752.6 | -1732.3 | -1317.7 |
| **NQO1** | **1D4A** | -1907.9 | -1684.9 | -1454.4 | -1769 | -1718.3 | -1391.3 | -1222.1 | -1269.8 | -1353.4 | -1568.7 | -1285.8 | -1504.2 | -1360.6 | -1985.1 | -1717.4 | -1531.2 | -1263.8 | -1501.3 | -1597.5 | -1271 |
| **ELOA2** | **AF-Q8IYF1-F1** | -1640.5 | -1632 | -1277 | -1746.8 | -1569.8 | -1566.6 | -1178.5 | -1331.5 | -1183.9 | -1632.9 | -1099.4 | -1400.4 | -1448.7 | -2117.6 | -1591.9 | -1597.9 | -1248.2 | -1460.5 | -1548.1 | -1269.8 |
| **APOA2** | **AF-P02652-F1** | -1849 | -1838.3 | -1266.5 | -1448.9 | -1254.9 | -1345.4 | -1353.9 | -1205.2 | -1296.9 | -1610 | -1202.2 | -1257.2 | -1347 | -1939.2 | -1526.8 | -1235.7 | -1277.4 | -1371 | -1121.3 | -1474.7 |
| **THBG** | **2XN6** | -1497.9 | -1438.3 | -1070.3 | -1564.7 | -1397.1 | -1143.9 | -994.4 | -1135.9 | -2058.5 | -1317.3 | -907.6 | -1040.3 | -1114.4 | -2623.4 | -1303.4 | -1362 | -1720.2 | -1316.5 | -1378.1 | -1335.9 |
| **MINT** | **7Z1K** | -1595.9 | -1629.7 | -1250.1 | -1551.5 | -1499.4 | -1332.1 | -1044.8 | -1212.7 | -1186.1 | -1294.2 | -1054.8 | -1323 | -1397.2 | -2097 | -1957.6 | -1128.7 | -1243 | -1284.1 | -1501.9 | -1001.7 |
| **A1BG** | **AF-P04217-F1** | -1850.8 | -1831.2 | -1149.7 | -1721.6 | -1417 | -1243 | -931.7 | -951.3 | -1195.1 | -1198.1 | -1306.7 | -1192.4 | -1185.1 | -2204.5 | -1229.2 | -1289.1 | -1220.4 | -1302.1 | -1454.9 | -1220.4 |
| **FETUA** | **AF-P02765-F1** | -1495 | -1514.1 | -1108.1 | -1613.6 | -1679.5 | -1311.3 | -991.3 | -1139.3 | -1320.2 | -1327.2 | -1071.3 | -1211.8 | -1168.9 | -1746.4 | -1423.5 | -1327.8 | -1269.1 | -1304.2 | -1470.7 | -1327.9 |
| **TM198** | **AF-Q66K66-F1** | -1825.5 | -1938.7 | NaN | NaN | -1598.5 | -1333.8 | -1119.5 | -1129.5 | -1551.6 | -1601.8 | -1232.7 | -1219.2 | -1343.6 | -2194.3 | -1770.9 | -1366.4 | -1364.7 | -1302.2 | NaN | -1914.1 |
| **RET4** | **5NU7** | -1654.4 | -1531.1 | -921.9 | -1470.3 | -1453.5 | -1212.2 | -852.6 | -1065.2 | -1174.7 | -1201.9 | -1272.6 | -1181.2 | -1178.5 | -1593.8 | -1359.8 | -1617.7 | -1208.9 | -1129 | -1227.9 | -1126 |
| **TTHY** | **7EJQ** | -1404.7 | -1528.1 | -927.6 | -1510.2 | -1305.7 | -1257.2 | -986.9 | -1024.6 | -1142.8 | -1426.5 | -1055.7 | -1202.3 | -1146 | -1637.9 | -1537 | -1238.2 | -1112 | -1242 | -1253 | -1241.3 |
| **VTDB** | **1KW2** | -1684.7 | -1480.6 | -1163.7 | -1343.1 | -1520.5 | -1271.3 | -1199.6 | -983.2 | -1195.3 | -1054.3 | -1199.6 | -1187.3 | -1240.9 | -1594.1 | -1063 | -1162 | -858.8 | -1288.2 | -1484.5 | -1127.5 |
| **UCHL1** | **4DM9** | -1701.8 | -1550.6 | -963.1 | -1812.3 | -1345.3 | -1199 | -997.8 | -878.9 | -1012.2 | -1249.7 | -891.9 | -1064.3 | -1120.1 | -1943 | -1210.1 | -1330.1 | -997.7 | -1300.4 | -1248.4 | -1093.8 |
| **ZA2G** | **1T80** | -1494.7 | -1633.4 | -976.3 | -1436.9 | -1188 | -1311.5 | -868.6 | -884.4 | -1144 | -1152.5 | -1061.7 | -984.1 | -1079.6 | -1558.1 | -1224.5 | -1240 | -948.3 | -1221.3 | -1178.6 | -1086.2 |
| **CBG** | **4C41** | -1349.5 | -1423.4 | -1022.7 | -1320.2 | -1324.4 | -1110.4 | -806.5 | -872 | -1871.9 | -1214.7 | -898.2 | -889.4 | -971.1 | -1752.2 | -1000.7 | -1133.4 | -1056.8 | -1174.9 | -1125.9 | -1013.6 |
| **MYH14** | **5I4E** | -1332.5 | -1355.1 | -895.6 | -1574.2 | -1186.8 | -1065.7 | -958.9 | -867.6 | -1159.4 | -1247.5 | -917.6 | -963.8 | -895.7 | -1692.6 | -1194.2 | -1102.9 | -1055.9 | -1121.5 | -1098 | -998 |
| **IC1** | **5DU3** | -1350.6 | -1319.3 | -1033.3 | -1372.8 | -1167.7 | -1196.9 | -764.2 | -886.3 | -1122.5 | -1013.6 | -818 | -1009.1 | -1162.9 | -1734.2 | -1306.8 | -1014 | -1017.3 | -1098.5 | -1043.5 | -982.3 |
| **CERU** | **4ENZ** | -1309.5 | -1294.7 | -950.9 | -1319.5 | -1080.5 | -946.4 | -923.2 | -954.4 | -1100.7 | -1176.3 | -996.6 | -1054.1 | -1035.3 | -1626.9 | -1057.1 | -1141.2 | -1010.6 | -1162.4 | -1039.3 | -1031.5 |
| **A2GL** | **AF-P02750-F1** | -1608.9 | -1476.9 | -782.9 | -1314.5 | -1057.7 | -1113.7 | -792.7 | -893 | -1098.2 | -926.1 | -903.7 | -889.4 | -880 | -1614.9 | -1363.2 | -1071.3 | -1148.5 | -992.5 | -1162.1 | -1021.3 |
| **LUM** | **AF-P51884-F1** | -1380.8 | -1284.4 | -964.8 | -1424.2 | -1320.3 | -967.2 | -720.3 | -816.3 | -1295.1 | -967.4 | -833.9 | -1030.6 | -970 | -1763.5 | -1175.9 | -978.9 | -981.1 | -1060.4 | -1241.4 | -851.8 |
| **A1AG1** | **3KQ0** | -1454.3 | -1210.5 | -914.1 | -1380 | -1255.7 | -1126.2 | -957.3 | -898.4 | -1036.1 | -1018.5 | -818.3 | -874.2 | -912 | -1398.2 | -1248.1 | -1211.7 | -820.8 | -1051 | -943.1 | -1014.5 |
| **ANGT** | **5M3Y** | -1396.6 | -1352.9 | -875.6 | -1212.6 | -962.8 | -988 | -868.3 | -865.3 | -1050.5 | -1063.8 | -868.8 | -959 | -935.5 | -1596.4 | -1212.6 | -1229.6 | -876.3 | -1020.5 | -861.7 | -939.5 |
| **APOH** | **6V06** | -1338.2 | -1244.4 | -757.2 | -1303 | -918.2 | -936.3 | -743.9 | -871 | -1137.8 | -1174.5 | -894.8 | -818.5 | -803.8 | -1444.7 | -1391.1 | -1052 | -1251.9 | -920.3 | -924.1 | -1052 |
| **ABCF1** | **5ZXD** | -1221.9 | -1246 | -724.7 | -1250.6 | -1165.2 | -1099.1 | -692.5 | -741.6 | -1113.2 | -943.3 | -796.4 | -819.6 | -862.3 | -1511.2 | -1141 | -1062.6 | -829.6 | -959.9 | -964 | -1062.9 |
| **A1AG2** | **3APU** | -1066.8 | -1066 | -811.1 | -1430.2 | -930 | -885.1 | -693 | -784.6 | -1022.4 | -978.6 | -819.4 | -725.6 | -748.7 | -1177 | -1031.3 | -1215.9 | -805.4 | -1054 | -806.3 | -966.6 |
| **TRFE** | **1A8E** | -1309.6 | -1091.7 | -753.5 | -1263.2 | -885.6 | -948.5 | -766.7 | -682.5 | -973.9 | -881.2 | -861.6 | -868.3 | -802 | -1362.3 | -1006.2 | -802.6 | -788.4 | -796.6 | -818.7 | -855.9 |

**Appendix 2:** The role of oocyte extract protein in triple-negative breast cancer reprogramming.


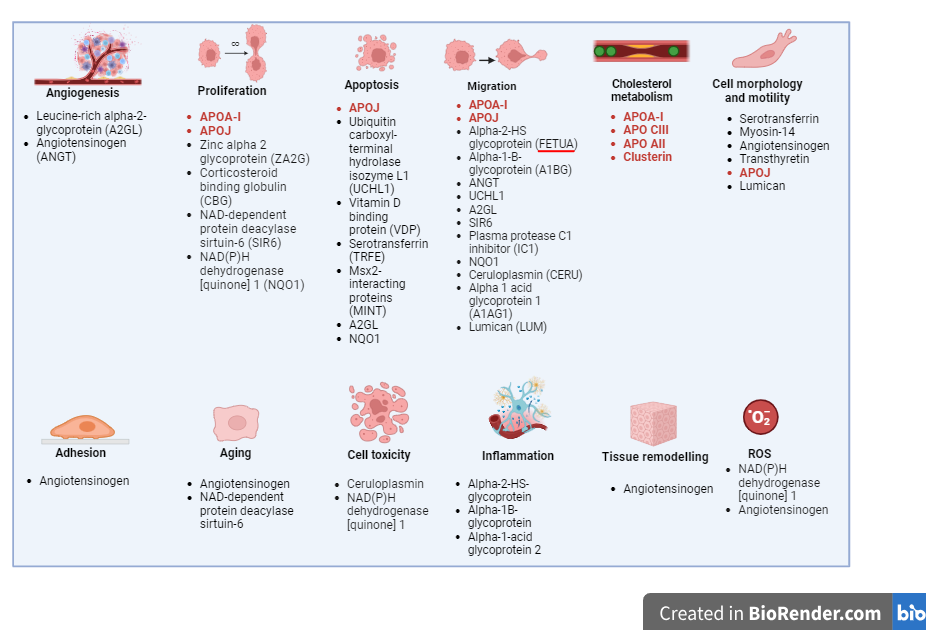

Supplement: Supplementary file 1 — Supplementary Information. [file 41598_2024_71522_MOESM1_ESM.docx]
